# American Dental Association

**Published:** 1893-04

**Authors:** 


### Reports of Society Meetings.

### AMERICAN DENTAL ASSOCIATION.

(Continued from page 207.)

### *Third Day.—Evening Session.*

MEETING called to order by the President, Dr. Walker.

The minutes of the morning session were read by the Assistant Secretary, the Secretary being absent on account of illness.

Section VI., Physiology and Etiology, announced the election of the following officers: H. A. Smith, Chairman, and L. E. Custer, Secretary.

Dr. Smith, chairman of Section VI., then reported as follows:

"At the last annual meeting your attention was called to the work undertaken by this Section in the examination of prehistoric crania. Satisfactory progress has been made in this investigation during the past year. Dr. J. J. Patrick will make a report of what has been accomplished, and also describe briefly the methods adopted in his investigation, and suggest a plan for the publication and preservation of the data obtained. As the work has proceeded the members of the Section have become more and more convinced of the great scientific value of this investigation, not only to ourselves, but to those who come after us; and in ordering these investigations the Association has shown the world that it is animated by the true scientific spirit.

"A paper has been submitted by Dr. Patterson, entitled 'The Effect upon the Tissues of the Oral Cavity produced by the Internal Administration of Certain Drugs,' and we recommend that it be read.

"I would report, as to the funds voted to the Section for the investigation just referred to, the following:

| | |
|---|---|
| "Balance on hand August 4, 1891 | $13.25 |
| Amount appropriated August 4, 1891 | 500.00 |
| Amount received by curator during the past year | 55.00 |
| Total | $568.25 |
| Amount expended by curator since August 4, 1891 | 225.05 |
| Leaving a balance of | $343.20 |

to be used for this work. Dr. Patrick has furnished the items of expenditure, and they have been audited by the proper officers of the Section."

Report received.

Dr. J. D. Patterson then read his paper on "The Effect upon the Tissues of the Oral Cavity produced by the Internal Administration of Certain Drugs," as follows:

The importance of this subject can scarcely be questioned by the observant dental practitioner, for he is almost daily brought into contact with certain diseased mouth conditions which are popularly believed to be the result of medication for certain constitutional or systemic conditions. This belief has been so long established, has been so fostered and taught by the physician as well as the dental practitioner, that it would be surprising if errors had not crept into or become coupled with the science of the question.

While listening to the history of cases as given by intelligent patients whose dental arches and surroundings have succumbed to disease, we find in very many cases that the inception of the trouble is firmly fixed in the patient's mind, and is believed to have been the result of certain medicines given for systemic effect, which have destroyed both the teeth and the gums and process which surrounded them. Such remarks as the following are very familiar: "My teeth were perfect until my physician gave me medicine during sickness, which salivated me," or, "I have taken so much iron that it has decayed my teeth." There are many other drugs which are thought to prejudice the integrity of the oral tissues, but the mercurial and iron preparations receive the burden of blame. The former, because the effect comes to the oral tissues alone through its internal administration, and because the belief in its ravages is so general, will receive attention in this essay to the exclusion of other drugs.

"The form in which mercury is given for the constitutional effect is commonly that of chlorides or iodides. A small dose, say one-tenth to one-twentieth of a grain of bichloride, is given every three or four hours until the desired effect is produced. The physiological action of mercury is tonic, purgative, alterative, antiphlogistic, and sorbefacient. The metal itself is inert, but by combination with the acids and fluids of the body becomes active, and is easily absorbed, passing into the blood from the stomach, the lungs, mucous membranes, or skin, in each case probably as an oxyalbuminate of mercury. In the blood its effects are tonic in small doses, but in quantity it produces impoverishment, diminishes the red blood-corpuscles, and disorders nutrition. It stimulates the glands of the body, especially the salivary, to the production of pathological secretions. In small doses, administered for a short time, the mercurial preparations are blood-tonics, increasing the number of red corpuscles and its general condition. Soon, however, they provoke waste by stimulating the lymphatics, and, if the small doses are continued, symptoms of so-called mercurial poisoning manifest themselves, and 'hydrargyrism,' commonly called 'salivation,' is exhibited. The symptoms of salivation or hydrargyrism are said to be a fetid breath, swollen and spongy gums, a blue line along the gum-margins, stomatitis, sore and loosened teeth, a metallic taste in the mouth. In extreme cases ulceration and sloughing occur around the teeth, and the jaw-bone is sometimes laid bare."

The foregoing is in brief a description of the effect upon the oral cavity of the giving of mercury for constitutional effect, as gleaned from medical and dental text-books, and especially from Potter's "Materia Medica and Therapeutics."

My purpose is to study briefly the situation, and to detail some practical experiments testing the truth of some of the usually accepted statements.

Some years ago, having been closely allied with physicians in the care of stomatitis resulting from mercurial poisoning, I began to doubt that the classic symptoms of ptyalism as found in the mouth were *per se* the result of mercury. My attention was first called to "*the blue line*," and text-books signally failed to enlighten me as to why it should be present. Inquiry among all classes of medical practitioners developed a wide difference of opinion about the *blue line*, that invariable symptom of salivation. They declared that it was there, that it was always present in salivation, and that was sufficient for them.

Now, in the earlier years of my practice, before I knew anything about mercurial poisoning, I had seen many "blue lines" around gum-margins as the result of a deposit of calcic matter, which disappeared under dextrous use of a scaler; and I asserted that the blue line was caused by calculus, and was not the result of mercury *per se.* I asked physicians to explain physiologically the presence of this blue line, and the only explanation was from an ignorant practitioner, who asserted that it was caused by the presence of finely divided metallic mercury gathered there from terminal vessels,—a thing utterly impossible, as mercury internally administered cannot reach the gum-tissue in any such form.

I then agreed to entirely obliterate in a single operation such blue lines in any patient brought to me, the patients to be chosen from those who were salivated by prolonged administration of mercurials. In this I was successful.

Finding that the "blue line" was a result of local conditions only, I then began to experiment upon the "inflamed and spongy gums" in salivated cases. I solicited cases of this kind, and stipulated to cure them without withdrawing the mercurial doses, if the physicians desired still to continue the mercury-giving. The result was perfectly successful. Under careful removal of all irritant matter, and the institution of perfect hygienic and antiseptic conditions, all swelling and ulceration, save that which was clearly syphilitic, in a few days would disappear; this too, as has been stated, without withdrawal or lessening of the dose of mercury. These experiments, which were carried on for about two years, were made possible from the fact that there were accessible a large number of patients suffering from the specific syphilitic taint under care of specialists.

The conclusions then reached, and which have since been verified, were that "the blue line," "the inflamed and spongy gums," and other of the more serious appearances were the result largely of local irritation, and not of the mercury; that, with proper care, these symptoms, which have been and are characterized as symptomatic of mercurial poisoning, may be entirely prevented.

In the condition of salivation we find the oral cavity unusually predisposed to the gathering of local irritant matter. There is an enormous, unusual secretion of vitiated saliva, and the precipitation of calcic matter is much increased. All the buccal secretions are unhealthy, and the condition demands more thorough hygienic care, and usually in such cases receives less; therefore the oral tissues fall a prey, not to the mercury, but to the intensely morbid local condition which the salivated condition has rendered possible. It is a condition similar in some particulars to that of the oral cavity in the case of pregnant women. The teeth fall a prey to decay in an unusual manner, not because they are softer at that time, but because "they then need more care and receive less." The fermentative processes are much more active, and the care is in inverse ratio to the needs.

Every observant practitioner must have noticed how closely the condition met with in irritation from calcic matter where there is no mercurial suspicion resembles the condition described in salivation. There is the blue line, the inflamed and spongy gums, sometimes covering the teeth, and ulceration of the gum-margins. The impoverished condition of the blood and the profuseness and character of all oral secretions constitute the chief difference. The unusual and malignant results sometimes occurring in salivation, and which do not appear in calcic irritation, may be safely laid to the unusual predisposition or the syphilitic condition which usually precedes the giving of mercurials. Indeed, Dr. Ringer, in his "Hand-Book of Therapeutics," says that the phenomena ascribed to mercurials are similar to those which will result from syphilis; and may it not, then, be true that the extraordinary symptoms referred to may be entirely due to the specific poison for which the mercury is given?

The whole question is one of interest and practical value to both dentist and physician. So long as the mercurials hold such an undisputed and merited position in therapeutics, it is of first importance to ascertain, if possible, just what are the evil results following their use.

It has been the purpose of this paper to show that some of the classic signs of mercurial poisoning which appear in the mouth are not symptomatic, but largely local; that the "blue line" is solely and only caused by calcic depositions; that the inflamed and spongy gums are resultant from the same cause, and from other irritant matter, such as fermenting particles of food-matter and mucus, and can be successfully prevented or cured by unusual attention to the removal of irritant matter and the establishment of thorough antiseptic conditions.

It would surely be a boon to diseased humanity if it were known that certain strict treatment of the mouth would prevent destruction of its tissues, when, on account of specific poison, it is desirable to continue potent doses of that prince of germ-killers, mercury. We believe this can be accomplished, and that much of the supposed ravages of mercury upon the tissues of the mouth may be safely ascribed to pure carelessness, ignorance, and neglect.

### DISCUSSION.

*Dr. W. W. Coon.*—I seriously question the statement that the blue line is always caused by the deposition of tartar around the necks of teeth. I took the impression of an edentulous jaw, in which there were several bluish deposits of pigmented matter. In several cases suffering from pyorrhœa, which had been under treatment for a long time, the necks of the teeth were entirely free from deposit; but if an instrument were passed in, and the gum held away from the root, the blue line would show.

*Dr. Fillebrown.*—I am quite in sympathy with the view Dr. Patterson takes of these cases which are often called ptyalism, but which I think depend upon other circumstances, we seldom seeing a case of real salivation. Many of the blue lines are caused by irritating substances under the edge of the gum. Lead is supposed to produce a blue line, but that does not always happen. I had a patient, a lady in perfect health, whose teeth and gums were in good condition, and the blue line was very marked around each tooth. That was twenty years ago, and the gums and teeth are still healthy. I find, as was said about germicides this morning, that there are many circumstances which control the phenomena in the human system. We cannot always tell what they are. I do not think any single disease or trouble causes it.

*Dr. Morgan.*—Had your patient ever used charcoal as a dentifrice?

*Dr. Fillebrown.*—I think not. I know the dentifrice she used.

*Dr. Kirk.*—I do not propose to dwell on the subject, but I should like to hear a description of the condition that was produced by the mercurial salivation. It is a rare occurrence. I know of a case where there was a distinct blue line around the gum-margin, more pronounced in front than at the posterior teeth. It was a case of pigmentation from charcoal, fine particles of it being embedded in the tissues themselves. It is like the case that Dr. Coon reported. In some of the teeth I could take an instrument and move the gum-tissues away from the tooth. The particles of charcoal were embedded there. The line was uniform and continuous, and the history of the case demonstrated that this patient had used charcoal as a dentifrice exclusively all her life. It gave a remarkable bluish cast to the gums.

*Dr. Smith.*—Reference has been made to the blue line which we occasionally observe about the necks of teeth, where there is no disease. In the few mouths of the African race that I have examined, I have found it amounting to a peculiarity that a blue line is present about their teeth. I have had in my practice one patient who had the blue line, and it was always suggested to me that she was not of the pure Anglo-Saxon race. If others have noticed it I would like to know. There was something besides this that impressed me that the woman, although she passed for white, was not so. I would like to ask Dr. Morgan if this is a characteristic of the African race?

*Dr. Morgan.*—It is one of their characteristics, and in the mixed races it very frequently happens. They are called blue-gum negroes, and there is a legend that the bite of a blue gum negro is death.

*Dr. Smith.*—Does this extend all around the teeth, or is it only anterior?

*Dr. Morgan.*—It is in lines; sometimes it is near the very margin of the gum; sometimes in patches around the teeth.

*Dr. Smith.*—In this case they were on the margin of the gum, and not continuous, but the pigmentation was more in patches.

*Dr. Hunt*, of Indianapolis.—While Dr. Patterson was reading his paper, the idea occurred to me that possibly in the majority of cases this blue line that we all talk about, but nobody gives a reason for, is usually due to congestion. That may have been in the minds of all who have spoken, but no one has said so. If it is due to congestion, as Dr. Patterson has said, it will undoubtedly be present whenever a low grade of irritation exists, whether it is caused by calculus or any other irritant, and is not produced particularly by the mercury, but possibly it is due to the greater deposition of salivary deposits because of the increased amount of saliva which is poured out while the medicine is being taken. I have never heard the cause explained. I should like to know what it is. Dr. Smith speaks of it as a pigment. If it is only a pigmentary deposit, it is something new to me.

*Dr. Gramm.*—I had a case of a patient in most excellent health who has had for a number of years the blue line, the typical blue line of mercurial poisoning. The writer stated that ulceration of the gums is due mainly and largely to neglect of the teeth or of the mouth under those conditions. The fact is, we have those very same conditions when there is no syphilitic taint present. When mercurials were used in days gone by they were not given for syphilitic conditions, but as a purgative when there was no trace of syphilitic poison. The writer compared also the degeneration of gum-tissue of the alveolus through mercurial action to the destruction of tooth-tissue during pregnancy. These cases to me seem not at all parallel, since the causes underlying the degeneration of tissue in one instance and the degeneration of tooth structure in the other are altogether different.

*Dr. Horton.*—I want to add a word to what has been said. From my observation I am satisfied that in nineteen out of every twenty, if not forty-nine out of every fifty cases, the local irritation and the appearance of the blue line can be traced to calculus deposit, more extensive in some than in others. In the case mentioned by Dr. Fillebrown, I am satisfied that a careful examination of that would reveal that the blue line was caused by minute particles of salivary deposit. I have seen very many cases affected that way. While it has been regarded among the medical authorities as a sure sign of the presence of mercurial irritation, I am satisfied that it was simply the increased amount of saliva, and the concentration of an inflamed condition there by the local irritation and by stopping the secretions and throwing them upon that part of the gum-tissue.

*Dr. Fillebrown.*—If Dr. Horton should have a patient eighteen or twenty years old, with perfectly healthy teeth and no other sign of anything present except a little blue line, he would not charge that to calculus, would he?

*Dr. Horton.*—It may be a deposit around the teeth. I have seen it in children very frequently.

*Dr. Fillebrown.*—That patient has been under my care for a long time, and Rigg's disease or calculus has never been exhibited.

*Dr. Kirk.*—I have been examining mouths more or less for fifteen years, and I have never seen a case of mercurial ptyalism. I would like the condition explained. I have asked Dr. Jack, and he has never seen a case either. I would like Dr. Horton to describe one of his many cases.

*Dr. Cravens.*—In some cases we have discoloration of the gum. It may not be blue, but it is certainly discolored, where the patients are in the habit of using soap for their teeth; the teeth will become coated with a film on account of the soap being used. The gentleman lost sight of the fact in talking of salivary calculus that the gum may be pushed away and still there will be the line. If salivary calculus will make a blue line, it would make it in the gum, and the gum would waste, because calculus may be deposited in any tissue. It might be in the gum-tissue.

*Dr. Barrett.*—I have seen a blue line along the gums at times, which was simply a surface deposit and was comparatively easily removed. The pigmentation or the coloring of it did not seem to penetrate to any particular depth. You could not wash it off, but it could be rubbed off. I never know to what to attribute it; I cannot conceive that mercurialism should cause a line along the gums. I have seen it cause a purple color in cases in which I have every reason to suppose it was mercurialization, not ptyalism. I did not suppose mercurialization would cause a blue line, but it will cause a pigmentation, not unlike that which you observe in necrosis of the bone.

*Dr. Patterson.*—My friend Dr. Gramm misapprehended the statement I made in regard to the condition known as salivation being analagous to the one in pregnancy. I merely compared the two, that in both cases the mouth needed more care and received less. I do not deny that there may be ulceration from hydrargyrism. There may be a low grade of irritation on account of the impoverished condition of the blood. The gums are pale; they are not blue, nor purple, but pale on account of the changed condition of the blood, and because the blood is so impoverished ulceration may be found in different parts of the body, but the inflamed and spongy gums that we find in cases that are called cases of salivation in a large degree are the result of local irritation. In the condition of ptyalism or mercurial poisoning the hydrargyrism is merely a condition of enormous secretion of all the fluids of the mouth, and of every part of the body, as far as that is concerned. The blood is impoverished, but as to the symptoms described by medical men and dental men in their text-books, I do not think they are at all indicative of the trouble. I have proved it many times. It is very difficult to find cases, but I was especially fortunate on account of being able to take advantage of many within two years which came from the hands of a specialist; he furnished them all to me.

*Dr. Morgan.*—I was reared in a country where mercurials and especially calomel were given very freely; and while I have not seen many cases since I have been a professional man, I have occasionally met some. I have seen it in all its stages. Not long since I was called to see a person who had a portion of the superior maxilla necrosed, which was the result of ptyalism. There was ulceration on the surface of the second superior molar, and the tooth was loose, and it took the crown of one of the permanent teeth with it.

*Dr. Patterson.*—How did you distinguish whether it was the mercury or the disease for which the mercury was given?

*Dr. Morgan.*—The physician told me.

*Dr. Patterson.*—How did he distinguish?

*Dr. Morgan.*—I don't know; I removed that portion of the maxilla. I had a case where I took out an inch and a half of the inferior maxilla, including one of the temporary teeth and the germs of two permanent teeth, which was the result of mercurialization. The boy had been taken sick, and had been given mercury very freely, and it was a severe case of salivation, as they called it, and that was the result. I have seen a great deal of ulceration in such cases. I have a patient living whose jaws cannot be opened.

We once gave, in the South, under the teachings of Cook, of the old Pennsylvania College, very large portions of mercury. I had a friend die recently to whom was administered within six or eight hours three hundred and sixty grains of calomel. They began with sixty grains; after a consultation they increased the amount to one hundred grains, and gave him three more doses. They administered it in that style all over that part of the country. I had an uncle die from an immense slough from the teeth.

As to the symptoms, the first is an unusual flow of saliva; then there is tenderness of the peridental membrane and soreness of the teeth until they become exceedingly tender; then there is a tenderness of the gum, and they lose to a considerable degree their density and their strength, and are easily wounded. I had a case not long ago from my homœopathic friends in the city of Nashville, and when I appealed to the lady, in one case, and told her she had been salivated, she said her doctor had as much right to give ten grains of calomel as any one else, and he did so, and her mouth got sore. In the other case it was denied; but there is a peculiar odor that you can at once detect. Of the consequences that follow and the changes that occur in the gums I am not familiar; because in these cases I have removed necrosed bone when it was necessary, and then referred them to the family physician for treatment. In nearly all these cases a loss of substance occurs, an absorption of the margin of the gums, and rapid absorption of the alveolar process. I had a friend die recently who was salivated many years ago, and rapid absorption occurred until all his teeth were much loosened and ready to drop out. He recognized that as a consequence of the salivation. There is a stage when you have that purplish appearance to which Dr. Barrett alludes, a simple chronic inflammation in which there is stagnation of the blood, where the blood is deteriorated, and you have the purplish line in the gum-tissue proper, not extending down on the maxilla beyond the gum-tissue. In that condition they are often exceedingly sensitive, and bleed very readily.

Subject passed.

Dr. Patrick then read a paper giving the result of his investigations relative to prehistoric crania, which had been ordered by the Association. After giving a number of statistics, he said,—

"When this work is finally completed, it will be an index to all the established museums in the country, containing crania that have been examined. There are, of course, many difficulties in an undertaking of this kind. Some of the crania in the Cambridge Museum were very ancient; they were gathered up out of confused heaps that vandals had produced by opening graves and stealing the silver and gold ornaments and pottery that had been buried with the mummies, throwing the ground and the skeletons and the crania all together. Huxley gathered these up, and as he gathered them he put his own number on, so in that condition they came in the possession of the Peabody Museum at Cambridge. When a private collection comes into the possession of such an institution, they leave the old numbers on of the original collector; then they put their own museum or catalogue number on; so in this work, where that occurs, the crania are in groups. Sometimes there are fifty under one number, carrying the original record number of the owner. I will also put that in the books which I am preparing, so if any question should arise in the future it can be verified immediately by referring to this appendix to our transactions, giving the number; all you need to do is to write to the curator. You can see on the diagram at once which side of the jaw it is; when this is complete it will be of great assistance, I am sure."

DISCUSSION.

*Dr. Peirce.*—There is nothing to discuss in the paper, except to emphasize the fact of the labor that has been done in the matter. I know some of the work, because some of it was under my supervision. I employed two young men, who were experts in that line, and after spending three afternoons with them, they pursued the work alone, and it took them nearly a month to go over twelve hundred skulls and give the different conditions, as noted in the table this afternoon. With regard to the measurements, I consider them of little use, although they have been taken with great accuracy; because the teeth vary so much that it is impossible to give figures that we can stand by. You cannot imagine the amount of work Dr. Patrick has had to do in order to give us this little summary. The value of this, when it is finished, will be very great, because it will give us a very definite idea of the condition of ancient skulls, and enable us to compare them with those more modern.

*Dr. Smith.*—I think when this work is complete that we are very likely to have some of our views disturbed in reference to the frequency of caries at the present time as compared with the ancients. Although many of them have not yet been tabulated, you will notice that all the lesions that are present in our race to-day were present in a goodly number in those prehistoric crania; and it would seem that if we are to draw any deductions from the report made, that caries was almost as prevalent at that day as it is now, and that caries of the teeth is not a result of civilization. I say we will probably have our views disturbed in that direction, and if we establish that fact, it will be of great value to us. Take the number of teeth tabulated; look at the large number of teeth lost from disease, because they were painful, showing that the lesions which cause the loss of the teeth to-day were present then. If we can only establish those facts it will be of incalculable value.

Dr. Patrick has very little faith that we can establish any very valuable facts from measurements. I was fearful that he would not make those measurements on account of his opinion, and it is very gratifying to find that he has done so, so that persons who hold this idea can prove it or disprove it. This work is so thoroughly done that for thousands of years it will not have to be repeated, and if any one doubts the conclusions that have been arrived at, he has the means at his disposal of going over the work and seeing for himself. It will be a monument not only to Dr. Patrick, but to the American Dental Association, even if it should be the only thing they have accomplished during their existence.

*Dr. Ottofy.*—I was very much pleased with the report as far as it has gone, and I am quite well satisfied from the statistics which have been read that when it covers the field which it eventually will, it will be the grandest thing the Association has ever undertaken. It is very interesting to notice the different conditions that have existed in prehistoric times. It is a great advantage in examining a cranium, because much that is covered by the gum-tissue can be seen at a glance, and I am in favor of continuing the work which Dr. Patrick is doing.

Subject passed.

*Dr. Kirk.*—I should like to report on the work of the newly-created Committee on State and Local Organization. It held a meeting to-day, and, pursuant to their instructions, I formulated a series of questions to cover as nearly as possible the field of dentistry that is covered by the sections of this body. I shall present them for the judgment of this society, so they may go out with the authority of the Association. It is the object of the committee to send out this series of questions to the different local organizations through the country, have them made part of the regular order of business, secure a report of the discussions from all the local societies, and have them referred to the proper sections of the American Dental Association; then the reports so received can be digested, and an abstract or an epitome of the results so obtained furnished to this body for discussion and for publication in their annual reports. The object is to get the consensus of the whole profession on certain topics of interest, and be able finally to formulate some definite principles of practice. We have formulated a series of ten questions, which are as follows. We have endeavored to cover the field as widely as possible.

1. Should examining boards have power to grant certificates of qualification to undergraduates?

2. Should immediate root-filling be practised while purulent conditions exist at the apex?

3. What are the best materials to enter into the composition of temporary fillings, to be retained for a minimum of three years?

4. What are the best methods for obtunding sensibility of the dentine by either local or general means? Should arsenic ever be used?

5. What are the best forms of partial lower dentures, and the methods for constructing the same?

6. Corrective dentistry; its present status. What are the simplest and most universally applicable forms of apparatus, and the most efficient retaining fixtures?

7. To what extent and under what conditions is the collar crown the cause of pericemental inflammation?

8. In cases of congested pulp, should arsenical application be made without preliminary treatment?

9. What are the advantages and disadvantages of the use of the matrix,—*a*, with gold; and *b*, with plastics?

10. The etiology of the pus formation?

If these questions meet with the approval of this body, I should be glad to have the same announced, and I make a motion that the circular, as read, shall be sent out by the committee with the authority of the Association. This is only for the coming year, of course. Different questions will be sent out annually.

Report received and motion carried.

Adjourned.

(To be continued.)

